# Purification of Actinium-225 from Thorium via Selective Precipitation

**DOI:** 10.3390/molecules31122144

**Published:** 2026-06-18

**Authors:** Steven J. Schultz, Sara L. Adelman, Guy H. Dutech, Michael E. Fassbender, Christopher D. Henning, Brian N. Long, Kristen A. Pace, Stosh A. Kozimor, Veronika Mocko, Thomas E. Shaw

**Affiliations:** 1 Los Alamos National Laboratory (LANL), P.O. Box 1663, Los Alamos, NM 87545, USA; sjschultz@lanl.gov (S.J.S.);; 2 Cyclotron Institute, Texas A&M University, 120 Spence Street, College Station, TX 77840, USA; 3 Department of Chemistry, Texas A&M University, 120 Spence Street, College Station, TX 77840, USA

**Keywords:** actinium-225, selective precipitation, thorium, nitrate salts, actinide separations

## Abstract

Numerous promising cancer treatments currently in clinical trials rely on the production and purification of actinium-225 (^225^Ac), an actinide with alpha emissions that can kill cancer cells via targeted alpha therapy. To enable ongoing and future studies, and to support anticipated future demand, it is necessary to increase the supply of ^225^Ac. High-energy proton irradiation of thorium metal (Th^0^_(s)_) is one of the leading production methods of ^225^Ac. This process requires the chemical separation of microscopic amounts (μg) of ^225^Ac from large quantities (>10 g) of thorium. Current methods to accomplish this thorium removal step can be slow, tedious, generate large quantities of radioactive liquid waste, and require very strict control of the processing conditions. To improve this separation, we investigated the ability of four nitrate salts (NH_4_NO_3_, KNO_3_, RbNO_3_, and CsNO_3_) to act as selective Th^4+^ _(*aq*)_ precipitation agents in the presence of ^225^Ac^3+^_(aq)_ in aqueous nitric acid to allow for their separation through a simple filtration. First, we used an automated separations platform to screen the ability of these nitrate salts to precipitate Th^4+^. We found the Th^4+^ precipitation yields and amount of precipitating agent needed to maximize this yield were dependent on the identity of the precipitating agent cation. Separation studies with ^225^Ac^3+^_(aq)_ and subsequent down-selection of the most promising Th^4+^ precipitating agents and conditions enabled us to develop its effective selective precipitation. We demonstrated that the separation was compatible with Th^0^_(s)_ quantities that can produce medically relevant amounts of ^225^Ac. We observed 99.9% of Th^4+^_(aq)_ could be removed via precipitation with KNO_3(s)_ in less than two hours in the presence of co-produced isotopes. Meanwhile, other experiments demonstrated that the ^225^Ac^3+^_(aq)_ recovery was > 97% at 1–10 g Th^0^_(s)_ scale.

## 1. Introduction

Actinides play an increasingly important role in many sectors, of which radiopharmaceutical therapy is one of the fastest growing [1,2]. Amongst the radionuclides that currently find wide-spread use for therapy (e.g., ^131^I, ^177^Lu, ^223^Ra) and imaging (e.g., ^99m^Tc, ^68^Ga, ^64^Cu, ^18^F) and amid those emerging as promising (^211^At, ^134^Ce, ^212^Pb to name only a few), actinium-225 (^225^Ac) stands out as having unique potential. It possesses nuclear properties that are ideal for targeted alpha therapy (TAT)—a mode of cancer therapy which utilizes the extreme cytotoxicity and localization of α-decay to destroy cancer cells, with dramatically reduced off-target effects relative to conventional therapies [3,4,5]. With four α-emissions, two β-emissions, and a half-life of 9.92 days [6,7,8], ^225^Ac is an isotope whose effectiveness has been demonstrated in numerous pre-clinical and clinical trials [9]. Already, two ^225^Ac-chelated complexes have progressed to phase III trials [10,11].

One obstacle slowing development of ^225^Ac-based TATs is the availability of ^225^Ac. There are several avenues being pursued to expand ^225^Ac supply, but each possesses a technical constraint. Previous in-depth discussions of ^225^Ac production methods and progress are available elsewhere [12,13,14]. In brief, the established route in the United States is generator production from ^229^Th to produce a high specific activity product of high radionuclidic purity [15,16,17,18]. However, this route is intrinsically source-limited: ^229^Th isolated from its uranium parent is rare and current quantities are insufficient to meet long-term clinical demand [19]. Direct cyclotron production of ^225^Ac via ^226^Ra(*p*,2*n*) is therefore an important near-term alternative, with mCi-scale (kBq-MBq) production demonstrated experimentally and recent development progressing toward clinically relevant batch sizes [20,21,22]. Its practical barriers, however, are dominated by the radioactive target itself: access to purified ^226^Ra, radon emanation, radiation-tolerant target fabrication and handling, and the necessity of near-quantitative Ra recovery and recycling. These same constraints extend to related ^226^Ra-based indirect production routes, including photonuclear production through ^226^Ra(γ,*n*) ^225^Ra and fast-neutron production through ^226^Ra(*n*,2*n*) ^225^Ra, both followed by β^−^ decay of ^225^Ra to ^225^Ac.

High-energy proton irradiation of natural Th offers the most scalable accelerator-based route because the target material is abundant and production can, in principle, reach curie-level batches at existing high-power proton facilities [23,24,25]. The tradeoff is chemical complexity and radiation dose control: irradiated Th targets contain a broad spallation/fission-product inventory, require heavily shielded remote processing, and co-produce small amounts of long-lived ^227^Ac, a long-term radiological liability that complicates clinical use [26,27,28,29,30]. Thorium spallation also produces ^225^Ra, which provides access to ^225^Ac free of ^227^Ac [26]; additionally, the spallation pathway may be utilized for ^213^Bi-based therapy through ^225^Ac/^213^Bi generators [26,31,32,33]. Thus, near-term expansion of ^225^Ac supply is likely to depend less on identifying a single dominant nuclear reaction than on solving target-processing problems that convert demonstrated nuclear yields into robust and clinically acceptable production workflows.

In the case of proton-irradiated thorium targets, chemical processing takes about one week. An important step of the purification process is the removal of gram-quantities of thorium metal (Th^0^_(*s*)_) target material, which is often accomplished by cation exchange chromatography [25]. Thorium removal using column chromatography is attractive because it is compatible with remote handling operations in hot cells required for handling highly radioactive material. Furthermore, it can be conducted on a time scale that maximizes the quantity of isolated ^225^Ac, and when performed correctly, cation exchange boasts high ^225^Ac recovery yields while removing large amounts of thorium.

Unfortunately, there are some challenges associated with this thorium removal procedure. It generates large amounts of radioactive liquid waste, requires strict pH control, and suffers from irreproducibility if the separation conditions are not rigorously maintained [34,35,36,37]. New thorium removal methods that reduce processing time and radioactive liquid waste, improve reproducibility, and lessen the operational burden on radiochemical processing technicians are therefore highly desirable for direct ^225^Ac production. In particular, avoiding pH-dependent manipulations and associated equipment, such as carefully calibrated pH-meter probes, could simplify processing and reduce opportunities for error resulting in poor separation efficiency. We hypothesized that accomplishing thorium removal via a selective precipitation of thorium from ^225^Ac-containing solutions could achieve these improvements and enable rapid and facile purification of ^225^Ac from proton-irradiated thorium targets.

Several methods have been developed to selectively precipitate thorium. While successful on relatively small scales (≤5 g of Th^0^), to the knowledge of the authors these methods (precipitation with hydroxide, peroxide, fluoride, oxalic acid, or iodic acid) [26,38,39,40,41,42] are not viable for larger scale ^225^Ac purification (≥10 g of Th^0^). Namely, the lack of selectivity for thorium, formation of difficult-to-filter solids, or the need to strictly control pH so far preclude their incorporation into larger-scale processes. Taking inspiration from these studies, we sought to develop a separation of actinium from thorium through selective precipitation that overcomes these obstacles. Solubility and crystallography work has demonstrated that tetravalent actinides (An^4+^) can form anionic species with the general formula [AnX_6_]^2−^ (where X = Cl^−1^, or NO_3_^−1^) that readily precipitate from aqueous solutions in the presence of alkali metal cations (M^1+^), presumably as M_2_AnX_6_ [43,44,45,46,47,48]. In hydrochloric acid, thorium typically forms cationic and neutral species, yet in highly concentrated nitric acid solutions thorium can form anionic species such as Th(NO_3_)_6_^2−^ [49,50,51,52,53,54]. On the other hand, ion-exchange chromatography separations have long exploited the fact that actinium does not form anionic species in strong mineral acids [55] and is stubbornly trivalent. Furthermore, previous spectroscopic studies identified that even in highly concentrated nitric acid solutions, the average speciation of Ac^3+^_(*aq*)_ is Ac(NO_3_)_2.3±1.7_(H_2_O)_8.3±5.2_^(0.7±0.5)+^ [56]. The relatively low charge density of Ac^3+^ is likely responsible for the lack of higher coordination of actinium by nitrate [57]. We postulated that we could leverage these chemical differences to achieve a separation of thorium from actinium by selectively precipitating M_2_Th(NO_3_)_6_ (when M = NH_4_^1+^, K^1+^, Rb^1+^, and Cs^1+^), while leaving Ac^3+^ dissolved in solution as a neutral or cationic species [51,52].

This study outlines a three-tiered approach to accomplish the previously mentioned goal. First we automated screening of MNO_3(*s*)_ as precipitation agents for Th^4+^_(*aq*)_ from nitric acid solutions on a small scale (ca. 70 mg Th_(*s*)_). Second, the most promising precipitation agents from the automated experiments were then tested for their ability to separate Th^4+^_(*aq*)_ from ^225^Ac^3+^_(*aq*)_ on a medium scale (ca. 1 g Th_(*s*)_). Third, the highest performing precipitation agents were tested on a scale that would be necessary to isolate medically relevant quantities of ^225^Ac, i.e., tens of grams of thorium and micrograms of ^225^Ac referred to as “large-scale” herein [26,58,59]. This tiered approach allowed us to quickly identify the best separation conditions. As a follow up, the optimized conditions were also evaluated in the presence of radioisotopes co-produced during the proton irradiation of thorium; qualitatively, medium- and long-lived species were not found to impact actinium–thorium separation.

Of the four nitrate salts tested, all demonstrated the ability to precipitate thorium from high concentrations of aqueous nitric acid. Moreover, two of the salts, KNO_3(*s*)_ and CsNO_3(*s*)_, achieved promising thorium–actinium separation. Ultimately, KNO_3_ was found to be the most effective selective precipitating agent under large-scale conditions. These findings have immediate relevance to the radiopharmaceutical industry and highlight how fundamental differences in actinide speciation can be impacted by counteraction identity, which in turn can be exploited for separations [14,58,59].

## 2. Results and Discussion

**Figure 1:**
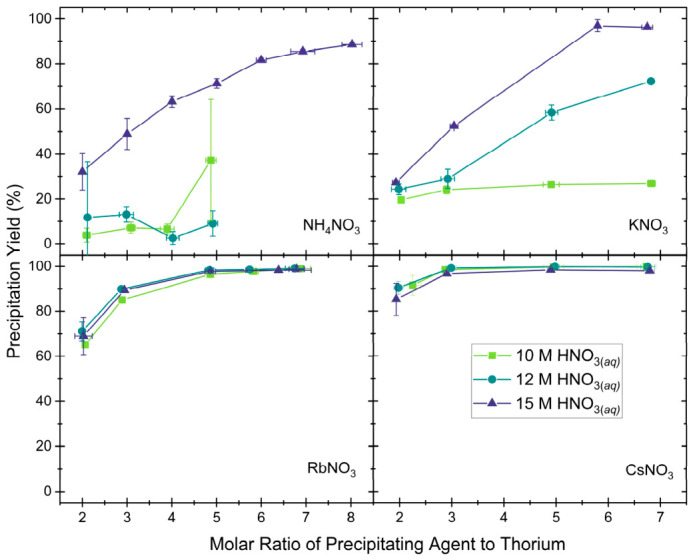
The thorium precipitation yields (%) from aqueous nitric acid solutions of Th^4+^_(*aq*)_ (0.3 M, 1 mL) with various precipitating agents, collected from automated experiments on the LANL Super Separator. Each precipitating agent was tested as function of cation identity (NH_4_^1+^, K^1+^, Rb^1+^, or Cs^1+^), the number of molar equivalents of precipitating agents relative to thorium, and nitric acid concentration. Lines between data points are to guide the eye and are not representative of a fit of the data. Each data point was collected in triplicate, and the error bars represent ±1σ.

To rapidly identify the most promising precipitating agent, its stoichiometry, and the nitric acid concentration required to maximize thorium precipitation from aqueous nitric acid solutions, four potential precipitating agents—NH_4_NO_3(*s*)_, KNO_3(*s*)_, RbNO_3(*s*)_, and CsNO_3(*s*)_—were screened using an automated separations instrument dubbed the Los Alamos National Laboratory Super Separator. This instrument automated small-scale (1 mL of 0.3 M Th^4+^_(*aq*)_) precipitations, filtrations, and analytical sample preparation—the results of which are summarized in Figure 1. The automation of these experiments permitted the rapid screening of more than 50 individual precipitating conditions in triplicate—close to 200 experiments. Quantification of Th^4+^_(*aq*)_ was achieved via inductively coupled plasma optical emission (ICP-OES) spectroscopy (details of this analysis are supplied in Section 3 Materials and Methods). All precipitating agents screened were capable of precipitating thorium at higher nitric acid concentrations, i.e., ≥10 M HNO_3(*aq*)_. This observation was consistent with the widely known nitric acid-dependent speciation behavior of Th^4+^_(*aq*)_, and with our prediction that Th^4+^_(*aq*)_ precipitation with alkali countercations would require anionic thorium species that dominate at higher HNO_3(*aq*)_ concentrations (>7 M) [38,53,60,61,62,63,64,65]. Increasing the HNO_3(*aq*)_ concentration above 10 M dramatically favored the formation of insoluble thorium complexes in the presence of NH_4_^1+^_(*aq*)_, K^1+^_(*aq*)_ countercations, while the thorium precipitation induced by Rb^1+^_(*aq*)_, and Cs^1+^_(*aq*)_ was essentially independent of HNO_3(*aq*)_ concentration (Figure 1).

Furthermore, the identity of the precipitating agent’s cation affected both the maximum thorium precipitation yield and the number of molar equivalents (relative to thorium) necessary to achieve this yield. KNO_3(*s*)_, RbNO_3(*s*)_, and CsNO_3(*s*)_ were all able to achieve a maximum thorium precipitation yield of > 99%. However, for NH_4_NO_3(*s*)_ the maximum thorium precipitation yield was only 88%. The number of molar equivalents necessary to achieve these maximum Th^4+^ precipitation yields followed the trend of Cs^1+^ (3) < Rb^1+^ (5) < K^1+^ (6) < NH_4_^1+^ (>8). It is interesting to note that this trend followed the general trend of alkali salt solubility noticed by Fanning, where solubility decreases with decreasing atomic number (i.e., Cs^1+^ < Rb^1+^ < K^1+^ < Na^1+^) [66]. These automated screening results confirmed that Th^4+^_(*aq*)_ could be precipitated under conditions relevant to the separation process after ^225^Ac production.

The optimized Th^4+^ precipitating conditions from the automated experiments were then tested for their ability to selectively precipitate Th^4+^_(*aq*)_ from solutions of dissolved thorium metal that also contained microcurie levels of the actinide of interest, ^225^Ac^3+^_(*aq*)_, and nanocurie levels of ^139^Ce*^n^*^+^_(*aq*)_, which was included to help predict the behavior and effects of co-produced lanthanides. First, a procedure for dissolving Th^0^_(*s*)_ in HNO_3(*aq*)_ was necessary to facilitate the precipitations. Typically, thorium dissolutions in HNO_3(*aq*)_ are significantly slower (>6 h) than those in HCl_(*aq*)_ and require relatively large quantities of HF_(*aq*)_ (up to 0.09 M) [67,68]. We found that the addition of (NH_4_)_2_SiF_6(*s*)_ to HNO_3(*aq*)_ for dissolution produced qualitatively fewer insoluble fines compared to the more commonly used HF_(*aq*)_, while still dissolving thorium metal in a short time. Ultimately, the optimized dissolution procedure used HNO_3(*aq*)_ (15.4 M, 150 mL per 10 g Th^0^_(*s*)_) with (NH_4_)_2_SiF_6(*aq*)_ (7 mM) at elevated temperature (75 °C) to quickly (≤2 h) dissolve tens of grams of Th^0^_(*s*)_ [69,70,71]. No loss of ^225^Ac^3+^_(*aq*)_ or ^139^Ce*^n^*^+^_(*aq*)_ during filtration was observed in the implementation of this procedure (Figure S1). Quantification of all radioisotopes utilized in this work was performed by gamma spectrometry on high purity germanium detectors (details in Materials and Methods).

**Figure 2:**
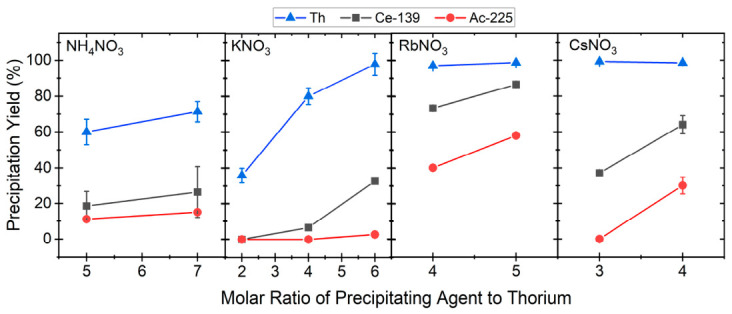
The precipitation yields of Th^4+^_(*aq*)_, ^225^Ac^3+^_(*aq*)_, and ^139^Ce*^n^*^+^_(*aq*)_ from aqueous solutions of Th^4+^_(*aq*)_ (0.3 M, 10 mL) freshly dissolved in HNO_3(*aq*)_ (15.4 M) and added ^225^Ac^3+^_(*aq*)_ and ^139^Ce*^n^*^+^_(*aq*)_ (radiotracer concentration) as a function of precipitating agent identity and stoichiometry. Lines between data points are to guide the eye and are not a representative fit of the data. Each data point was collected in duplicate, and the error bars represent ±1σ.

Solutions that contained Th^4+^_(*aq*)_ (~0.3 M), ^225^Ac^3+^_(*aq*)_ (~0.5 µCi/mL) and ^139^Ce^3+^_(*aq*)_ (~10 nCi/mL) obtained from Th^0^_(*s*)_ dissolution were tested for thorium removal on a medium scale (ca. 1 g Th^0^_(*s*)_). Precipitation of Th^4+^_(*s*)_ was achieved under the experimental conditions identified by the automated screening experiments described above (Figure 1). However, the ability of each precipitating agent to do so selectively, i.e., precipitate thorium as a solid and leave ^225^Ac^3+^_(*aq*)_ in solution, did not follow a clear trend across the evaluated precipitation agents (Figure 2). Among all conditions tested, NH_4_NO_3(*s*)_ performed the poorest. Since only modest Th^4+^ precipitation yield (71.3%) could be achieved with as many as seven molar equivalents (with respect to thorium), and since significant co-precipitation of ^225^Ac^3+^_(*aq*)_ (15.1%) and ^139^Ce*^n^*^+^ (26.4%) occurred, we did not pursue this precipitating agent further. Additionally, RbNO_3(*s*)_, despite having excellent Th^4+^ precipitation yields, deleteriously co-precipitated ^225^Ac^3+^_(*aq*)_ and ^139^Ce*^n^*^+^_(*aq*)_. The two most promising conditions, achieving a high Th^4+^ precipitation yield and high retention of ^225^Ac^3+^_(*aq*)_ in solution, were KNO_3(*s*)_ (six equivalents) and CsNO_3(*s*)_ (three equivalents). Both of these conditions co-precipitated comparable amounts of ^139^Ce*^n^*^+^ (32.8% and 37.1%, respectively).

While a detailed structural analysis of the thorium precipitate formed using each alkali nitrate salt and its ability to co-precipitate Ac^3+^ and Ce*^n^*^+^ is beyond the scope of this study, it is useful to note that characterization of the insoluble products formed under conditions relevant to precipitation experiments via single-crystal and powder X-ray diffraction reported herein were consistent with the composition of products reported by Soderholm (Table S1 and Figures S6–S8). Namely, we isolated M_2_Th(NO_3_)_6_ (M = NH_4_^1+^, Cs^1+^, Rb^1+^) when NH_4_NO_3(*s*)_, CsNO_3(*s*)_, or RbNO_3(*s*)_ was added to Th(NO_3_)_4_·xH_2_O_(*aq*)_ and a mixture of products that included K_3_[Th(NO_3_)_6_](NO_3_)(HNO_3_)_3_·3H_2_O when KNO_3(*s*)_ was the precipitating agent. Future studies on whether M_2_Th(NO_3_)_6_ complexes are better than M_3_[Th(NO_3_)_6_](NO_3_)(HNO_3_)_3_·3H_2_O at trapping cations such as Ac^3+^ and Ce*^n^*^+^ during their precipitation from HNO_3(*aq*)_ solutions are necessary to elucidate the mechanism behind these observations. Additionally, the tendency of Ce^3+^ to oxidize to Ce^4+^ in the presence of strong oxidants provides another possible pathway for cerium precipitation via the formation of M_2_Ce(NO_3_)_6_ [45,72,73]. Follow-on work characterizing the oxidation state of cerium under these conditions is needed to understand the co-precipitation behavior of cerium.

After the identification of KNO_3(*s*)_ and CsNO_3(*s*)_ as effective agents for the selective precipitation of Th^4+^_(*aq*)_ from ^225^Ac^3+^_(*aq*)_, research efforts shifted to focus on the evaluation of thorium dissolution and selective precipitation on a large scale. We dissolved ^nat^Th^0^_(*s*)_ (10 g) in the presence of ^225^Ac^3+^_(*aq*)_ and ^139^Ce*^n^*^+^_(*aq*)_. To the subsequent solutions, ca. 0.29 M Th^4+^_(*aq*)_, 145 mL, either KNO_3(*s*)_ (25 g, 250 mmol) or CsNO_3(*s*)_ (25 g, 130 mmol) was added. In each case, after the addition of the precipitation agent and mixing for 1 h, a white precipitate was collected via filtration. The collected solids were washed with a solution of the corresponding precipitating agent (2 M KNO_3(*aq*)_ or 1 M CsNO_3(*aq*)_) in HNO_3(*aq*)_ (15.4 M), see Figures S2 and S10. The concentrations of Th^4+^_(*aq*)_, ^225^Ac^3+^_(*aq*)_, and ^139^Ce*^n^*^+^_(*aq*)_ present in these filtrates were compared to their ingoing concentrations and are presented in Table 1. Both precipitating agents removed the majority of the initial Th^4+^_(*aq*)_ from solution (i.e., percent isolated in the filtrate < 5%). However, at this scale, KNO_3(*s*)_ clearly outperformed CsNO_3(*s*)_ from a separation standpoint, as only 2.6 ± 2.0% of the ^225^Ac^3+^_(*aq*)_ was lost through the process when KNO_3(*s*)_ was used as precipitating agent, while 22.4 ± 0.3% was lost when CsNO_3(*s*)_ was used for Th^4+^_(*aq*)_ as a precipitation agent. We speculate the reason behind the lower percent recovery of ^225^Ac^3+^_(*aq*)_ with CsNO_3(*s*)_ compared to KNO_3(*s*)_ is due to the structural and composition differences between the insoluble products formed. Confirmation of whether these structural and compositional differences in thorium nitrate complexes ultimately lead to less co-precipitation of Ac^3+^_(*aq*)_ would require structural studies beyond the scope of this work.

**Figure 3:**
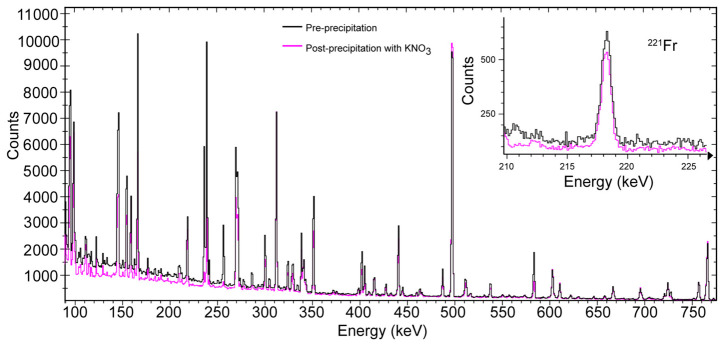
Qualitative comparison of the gamma spectra of (black trace) an aliquot of Th^0^_(*s*)_ (10 g) dissolved in HNO_3(*aq*)_ (15.4 M) with irradiated target material and (magenta trace) the resulting filtrate following selective Th^4+^_(*aq*)_ precipitation with 6 molar equivalents of KNO_3(*s*)_ (6 molar equivalents with respect to Th). Spectra of the wash steps which recovered some additional ^225^Ac^3+^_(*aq*)_, are not included.

To investigate the potential impact of isotopes co-produced with ^225^Ac during thorium spallation on the separation Ac^3+^_(*aq*)_ from Th^4+^_(*aq*)_ via selective precipitation, each large-scale thorium precipitation process was repeated in the presence of dissolved irradiated thorium target aliquot received from Oak Ridge National Laboratory. Owing to the age of this irradiated material (ca. 60 d from end of bombardment), we could not capture the impact of shorter-lived (i.e., with half-lives less than 10 d) radionuclides on these separations. However, we still find these studies insightful because they tested the efficacy of the procedure that separated thorium from actinium in the presence of at least 45 relevant co-produced radionuclide contaminants (Figures S3–S5). In these studies, we monitored Ac^3+^_(*aq*)_ by the presence of its ^221^Fr progeny by gamma spectrometry (218 keV photopeak; Figure 3), and Th^4+^ was quantified using ICP-OES. Precipitation of Th^4+^ with KNO_3(*s*)_ in the presence of co-produced isotopes proceeded exceptionally well, with 99.975 ± 0.001% of the Th^4+^ precipitated and qualitatively all of the ^225^Ac^3+^_(*aq*)_ recovered in the filtrate and washes (Figure 3 inset).

Performance of CsNO_3*(s*)_ was also impressive, with 98.29 ± 0.03% precipitated while the majority of the Ac^3+^*_(aq_*_)_ stayed in the filtrate (Figure S3). Qualitatively, we observed that most of the co-produced radioisotopes either followed Ac^3+^*_(aq_*_)_ during the Th^4+^ precipitation or bifurcated between the filtrate and precipitate (Figure S5). While CsNO_3(*s*)_ showed some promise as a selective precipitation agent for the recovery of Ac^3+^_(*aq*)_ from Th^4+^_(*aq*)_, KNO_3(*s*)_ was an exceptional selective precipitation agent that permitted qualitatively excellent Ac^3+^ recovery while removing 99.9% of the initial Th^4+^ in the presence of more than 45 co-produced radioisotopes.

Relative to citrate-based cation exchange for thorium removal, our results indicate that the KNO_3(*s*)_ precipitation method provides measurable improvements in processing time, robustness, liquid inventory, and suitability for remote operation, while maintaining high ^225^Ac recovery. At the 10 g Th_(*s*)_ scale, the precipitation process uses the thorium target solution directly obtained from nitric acid dissolution. KNO_3(*s*)_ is added to the high-nitrate Th^4+^_(*aq*)_ solution and mixed for 60 min (minimum time 30 min), followed by filtration to separate solids in ca. 5–10 min. The precipitate is then washed up to three times to recover entrained ^225^Ac, requiring ca. 10 min per wash, giving a total thorium-removal time of approximately 85–100 min after dissolution (Figure S10). This time-efficient and robust procedure for separating thorium from actinium can be completed in under 2 h with remote handling manipulations in hot cells and is amenable to routine production campaigns for a short-lived isotope such as ^225^Ac.

In contrast, cation-exchange methods require matrix conversion from hydrochloric acid media to a citrate-complexed, tightly pH-controlled solution, followed by column conditioning, loading, washing, and ^225^Ac elution. Radchenko et al. used a 75 mL pH 2 citrate load and 20 mL citrate wash followed by HNO_3(*aq*)_ elutions for only 0.5 g Th_(*s*)_. Fitzsimmons et al. identified pH 1.5–2.0 as optimal and used a 37 bed-volume (BV) rinse and elution sequence after recognizing that earlier 63.3 BV citrate/13.3 BV HNO_3(*aq*)_ workflows were excessive in time and waste generation [34,36]. When the volumes for the cation-exchange method are linearly normalized to 10 g Th_(*s*)_, 1–2 L of liquid waste is generated. By comparison, selective precipitation generates only ca. 205–210 mL of liquid with thorium isolated as a compact solid precipitate. Finally, the KNO_3(*s*)_ precipitation method is more robust as the separation is driven by Th^4+^_(*aq*)_ nitrate speciation in concentrated HNO_3(*aq*)_, avoiding the narrow pH window required for citrate complexation of Th^4+^_(*aq*)_ and subsequent separation from ^225^Ac^3+^_(*aq*)_ on cation-exchange resin.

Studies are ongoing to evaluate the integration of selective precipitation with subsequent purification steps as well as the fate of shorter-lived co-produced radionuclides during the precipitation operation.

## 3. Materials and Methods

**General considerations.** Caution! Natural thorium (^232^Th, t_½_ = 1.40 × 10^10^ y), actinium-225 (^225^Ac, t_½_ = 9.920 d), cerium-139 (^139^Ce, t_½_ = 137.64 d) [74] and their progeny constitute serious health threats because of radioactive decay. Hence, all experiments that involved manipulation of these radionuclides were conducted in radiological buffer areas that contained HEPA-filtered hoods, continuous air monitors, and monitoring equipment appropriate for α-, β-, and γ-particle detection. Entrance to laboratory spaces was controlled with monitoring instruments for α-, β-, and γ-emitting isotopes and full-body personal contamination-monitoring stations. Water (H_2_O, RICCA Chemical Company, Arlington, TX, USA, ACS reagent grade Type I), aqueous nitric acid (HNO_3_(*aq*), Fisher Scientific, Pittsburgh, PA, USA, Optima^®^ grade, 68%, 15.4 M), thorium(IV) nitrate hydrate (Th(NO_3_)_4_·*x*H_2_O, Strem Chemicals, Newburyport, MA, USA, 99.8%), ammonium nitrate (NH_4_NO_3_, Sigma-Aldrich, St. Louis, MO, USA, ≥99.5%), cesium nitrate (CsNO_3_, Thermo Fisher Scientific, Waltham, MA, USA, ≥99.8%), potassium nitrate (KNO_3_, Sigma-Aldrich, St. Louis, MO, USA, >99.0%), rubidium nitrate (RbNO_3_, Sigma-Aldrich, St. Louis, MO, USA, 99.7%), and ammonium hexafluorosilicate ((NH_4_)_2_SiF_6_, Acros Organics, Geel, Belgium, 99.999%), were obtained commercially and used as received. Actinium-225 was provided by the National Isotope Development Center (Oak Ridge, TN, USA) and the Department of Energy’s Isotope Production Program (Washington, D.C., MD, USA) as a dry nitrate residue containing a small amount (ca. 1–2%) of actinium-227. Natural thorium metal was obtained from Sigma Division at Los Alamos National Laboratory (Los Alamos, NM, USA). Cerium-139 in HCl_(*aq*)_ (0.1 M) was obtained as a byproduct of cerium-134 production from Department of Energy’s Isotope Production Program. No attempt was made to characterize the oxidation state of cerium-139. Dissolved irradiated thorium target material was provided by the National Isotope Development Center and the Department of Energy’s Isotope Production Program as an aqueous solution in HCl_(*aq*)_/HF_(*aq*)_ (10 M/trace) or HCl_(*aq*)_/HNO_3(*aq*)_ (0.2 M/0.1 M) solution, respectively. For both the dissolved irradiated Th^4+^_(*aq*)_ target and ^139^Ce*^n^*^+^, a matrix conversion to HNO_3(*aq*)_ (15.4 M) was performed by evaporating the samples to dryness and re-constituting the residue in concentrated nitric acid prior to use in separation experiments. This process was performed three times to ensure complete conversion. Mixing of solutions in non-automated experiments was performed on a VWR^TM^ Digital Vortex Mixer (500–3200 rpm) from VWR (Radnor, PA, USA).

**Gamma spectrometry.** All gamma spectrometry was performed on ORTEC^®^ P-type coaxial high purity germanium (HPGe) detectors: GEM 30 P4-76 (61.9 mm diameter; 43.4 mm length; 1 mm Al window (0.7 mm Ge/Li outer dead layer, 0.3 µm Ge/B inner dead layer); resolution (FWHM) of 1.75 keV at 1333 keV) and GEM 10 P4-70 (47.4 mm diameter; 43.8 mm length; 1 mm Al window (0.7 mm Ge/Li outer dead layer, 0.3 µm Ge/B inner dead layer); resolution (FWHM) of 1.72 keV at 1333 keV) both from AMETEC ORTEC, Oak Ridge, TN, USA. The HPGe detectors were housed in lead shielding, cooled with liquid nitrogen (Ortec Möbius cryostat system 805709) and coupled to DSPEC 50 (GEM 30 P4-76) and DSPEC 502 (GEM 10 P4-70) multichannel analyzers, respectively (AMETEC ORTEC, Oak Ridge, TN, USA). The efficiency of the detector was calibrated in the energy interval of 46.5 keV to 1836.1 keV for a uniform geometry (5 mL aqueous solution in a 20 mL plastic liquid scintillation vial) using a mixed radionuclide reference solution (containing ^210^Pb, ^241^Am, ^109^Cd, ^57^Co, ^139^Ce, ^203^Hg, ^113^Sn, ^85^Sr, ^137^Cs, ^88^Y, and ^60^Co) traceable to the National Institute of Standards and Technology purchased from Eckert and Ziegler (Valencia, CA, USA). Daily calibration checks were performed with a ^152^Eu source (Eckert and Ziegler) in the same geometry. Counting dead time was maintained below 10%. Resulting spectra were analyzed with GammaVision Gamma Spectroscopy software version 8.10.02. ^139^Ce quantification was achieved through measurement of its 165 keV peak (79.9%). ^225^Ac quantification was achieved from the activities of its progeny, ^221^Fr (218 keV, t_1/2_ = 4.9 min) and ^213^Bi (440 t_1/2_ = 45.59 min) after waiting 24 h post-separation to establish secular parent–progeny equilibrium. All measurements for a given experiment were made on the same detector, at the same position, and in an identical geometry.

Complex gamma-ray spectra of irradiated thorium target material were assessed for isotopic composition by fitting with the python package Curie 0.0.32 (released under MIT License, Copyright 2024 Jonathan Morrell) [75]. Isotopes identified in the matrices incorporating irradiated target material included ^111^Ag, ^110m^Ag, ^140^Ba, ^213^Bi, ^206^Bi, ^144^Ce, ^141^Ce, ^139^Ce, ^136^Cs, ^132^Cs, ^221^Fr, ^130^I, ^124^I, ^140^La, ^96^Nb, ^95^Nb, ^147^Nd, ^233^Pa, ^230^Pa, ^214^Pb, ^212^Pb, ^211^Pb, ^208^Pb, ^148m^Pm, ^206^Po, ^223^Ra, ^86^Rb, ^219^Rn, ^103^Ru, ^126^Sb, ^125^Sb, ^124^Sb, ^125^Sn, ^117m^Sn, ^123m^Te, ^129m^Te, ^121m^Te, ^231^Th, ^227^Th, ^209^Tl, ^208^Tl, ^207^Tl, ^202^Tl, ^99m^Tc, ^72^Zn, and ^95^Zr. While quantification of the predominant co-produced isotopes falls outside of the scope of this paper, it is worth noting that the fitting parameters determined so far demonstrate contamination of the 440 keV peak of bismuth-213 with ^202^Tl (t_1/2_ = 12.31 d; 439.51 keV, 0.915 b.r.). This has led the authors to suggest reliance on the 218 keV peak of ^221^Fr for ^225^Ac quantification in systems possessing irradiated thorium target material.

**Inductively coupled plasma optical emission spectrometry (ICP-OES).** The quantification of thorium was performed utilizing an Avio 500 inductively coupled plasma optical spectrometer (PerkinElmer, Shelton, CT, USA). The instrument features a dual-view optical configuration and a vertical torch design, which was operated in axial viewing mode to maximize sensitivity and lower detection limits for the actinide analyte. Plasma generation was sustained using a solid-state radio-frequency generator operating at a forward power of 1500 W. The sample introduction system consisted of a TFE Tracey Spray Chamber (50 mL) coupled to a PTFE MiraMist Nebulizer (0.2–2.5 mL/min), both from PerkinElmer (Shelton, CT, USA), to optimize aerosol residence times and resist HF corrosion. Sample delivery was regulated via a peristaltic pump equipped with standard PVC pump tubing. Argon gas (99.999%) served as the plasma, auxiliary, and nebulizer gas flow medium. The instrument was controlled and data processed using the proprietary Syngistix for ICP software platform (v. 5.5.0.1274). Thorium emission lines were selected based on high net signal intensity, peak symmetry, and the absence of spectral overlaps within the nitric acid matrix. The primary analytical wavelength monitored was 283.730 nm due to its superior signal-to-noise ratio in axial plasma viewing modes. Secondary lines at 339.204 and 401.913 nm were concurrently acquired to cross-verify analytical accuracy and flag potential uncompensated matrix spectral interferences. Background corrections were applied via automated two-point off-peak background subtraction with the Syngistix software (v. 5.5.0.1274).

**Limit of Detection and Limit of Quantification.** The Limit of Detection (LOD) and Limit of Quantification (LOQ) for the quantification of thorium via ICP-OES were determined using Equations (1) and (2).

LOD = 3 × (*s_b_*/*m*) (1)

LOQ = 10 × (*s_b_*/*m*) (2)

Here, *s_b_* was the error in the *y*-intercept for the corresponding linear regression constructed using four calibration points and *m* was the slope of this line [76,77]. 

**Calculation of percent precipitation**. The amount of an analyte precipitated was calculated as a precipitation yield using Equation (3):

(3) $$Precipitation\;Yield=\;100\times \frac{\left({\left[M\right]}_{i}\;-{\;\phi \;\left[M\right]}_{f}\right)}{{\left[M\right]}_{i}}$$

where [*M*]*_i_* was the concentration of a given analyte prior to precipitation, [*M*]*_f_* was the concentration of the analyte in the filtrate post precipitation and filtration, and *ϕ* was the dilution correction factor.

**Automated screening of thorium precipitating agents (small ~70 mg Th scale).** The efficacy of nitrate salts to precipitate thorium from solutions with variable acid and precipitating agent concentrations was investigated using the Los Alamos National Laboratory Super Separator (see Figure S9), a custom-designed Big Kahuna automated instrument from Unchained Labs Inc. (Pleasanton, CA, USA). The Super Separator automates nearly every step associated with a generic selective precipitation. The instrument is equipped with liquid handling, which achieves a liquid dispensing accuracy of ±10 μL with a precision within ±5% up to 10 mL, and solid dispensing, which can dispense as little as 2 mg of solids with an accuracy and precision of ±2%. The time, temperature, and mixing speed of experiments were controlled. Two software packages, Library Studio and Automation Studio (both version 9.2.40820.1), were used to design and execute the selective precipitation workflows described below, both of which were provided by Unchained Labs Inc. to interface with the LANL Super Separator.

In a typical screening experiment, stock solutions of Th^4+^_(*aq*)_ (ca. 0.3 M) were prepared manually by adding Th(NO_3_)_4_·*x*H_2_O (3.6 g, 7.5 mmol) to a volumetric flask (25 mL) dissolved and filled up with HNO_3(*aq*)_ (10, 12, or 15.4 M). The LANL Super Separator dispensed the solid precipitating agents from glass vials (4 mL, Wheaton, Millville, NJ, USA) equipped with a powder-dispensing valve (Unchained Labs Inc.) into glass vials (4 mL, Wheaton) and recorded the mass on an analytical balance (Unchained Laboratories). To these solid precipitating agents, the Super Separator added the stock solution of Th(NO_3_)_4_·*x*H_2_O (0.3 M, 1 mL, Rainin) in a given concentration of nitric acid (10, 12, or 15.4 M). The resulting mixtures were vortexed (800 rpm, 1 h, Big Bear Orbital Shaker, Santa Clara, CA, USA) at room temperature. The mixtures were then allowed to settle (10 min) and transferred to the chamber of a syringeless filter (polypropylene, Whatman Mini-UniPrep, Wilmington, DE, USA). These mixtures were manually filtered (PVDF filter media, Whatman Mini-UniPrep, 0.2 mm). The Super Separator then dispensed an aliquot of the filtrate (20 mL, Eppendorf, Enfield, CT, USA) and performed a dilution (by a factor of 1000) with HNO_3(*aq*)_ (0.1 M) for analysis by ICP-OES to determine the final concentration of Th^4+^_(*aq*)_ ([Th]*_f_*). Aliquots of the thorium nitrate stock solutions (20 mL, Eppendorf) were taken by the Super Separator and diluted by a factor of 1000 with HNO_3(*aq*)_ (0.1 M) for analysis by ICP-OES to determine the initial concentration of Th^4+^_(*aq*)_ ([Th]*_i_*). All measurements were performed in triplicate.

**Thorium metal dissolution.** In a typical dissolution, thorium metal (6.6 g, 28 mmol), HNO_3(*aq*)_ (15.4 M, 100 mL), and (NH_4_)_2_SiF_6_ (0.096 g, 0.56 mmol) was added to an Erlenmeyer flask (250 mL, PYREX^®^). To this mixture, ^225^Ac^3+^_(*aq*)_ ca. 5 μCi (185 kBq) and ^139^Ce*^n^*^+^_(*aq*)_ 0.1 μCi (3.7 kBq) in HNO_3(*aq*)_ (15.4 M) were added. The resulting mixture was heated to approximately 75 °C and stirred until gas evolution visibly ceased (ca. 2 h). The resulting mixture was allowed to cool to room temperature and vacuum filtered (0.22 µm PVDF–250 mL, Durapore Stericup^®^ Quick Release, Darmstadt, Germany) to remove any undissolved white residues, presumably small quantities of thorium oxide [78,79]. The filter was washed with Type I H_2_O (10 mL) and analyzed via γ-spectrometry to confirm complete recovery of ^225^Ac^3+^_(*aq*)_ in the filtrate (Figure S1). The concentration of dissolved Th^4+^_(*aq*)_ ([Th]*_i_*) was determined by ICP-OES by averaging the measured concentration of aliquots of the filtrate (3 × 50 mL) diluted with HNO_3(*aq*)_ (0.1 M) by a factor of 10,000. Additionally, three undiluted aliquots (5.00 mL) of the dissolved thorium solution were taken and analyzed via γ-spectrometry to determine the initial concentrations of ^225^Ac^3+^_(*aq*)_ and ^139^Ce*^n^*^+^_(*aq*)_ ([Ac]*_i_* and [Ce]*_i_*, respectively). Following analysis, these aliquots were returned to the stock solution.

**Separation of thorium from actinium-225 and cerium-139 via selective precipitation (*medium* ca. *1 g scale of dissolved thorium metal*).** Aliquots (10 mL) of thorium metal dissolved in HNO_3(*aq*)_ (15.4 M) with trace (NH_4_)_2_SiF_6(*aq*)_ (ca. 7.5 mM) following the procedure given above were dispensed into HDPE scintillation vials (20 mL, WHEATON^®^). To these solutions, either NH_4_NO_3(*s*)_ (1.2–1.7 g, 15–21 mmol), KNO_3(*s*)_ (1.2–1.8 g, 12–18 mmol), RbNO_3(*s*)_ (1.8–2.2 g, 12–15 mmol), or CsNO_3(*s*)_ (1.75 g, 9 mmol) were added as solids. The resulting suspensions were mixed (550 rpm, 1 h) and the white precipitates were removed via filtration (Whatman 0.45 µm PTFE syringe filter).

Aliquots (5.00 mL) of each filtrate were taken and analyzed via γ-spectrometry to determine the final concentrations of ^225^Ac^3+^_(*aq*)_ and ^139^Ce*^n^*^+^_(*aq*)_ ([Ac]*_f_* and [Ce]*_f_*, respectively). Additional aliquots of the filtrate (3 × 50 mL) were taken and diluted with HNO_3(*aq*)_ (0.1 M) by a factor of 100–1000. These diluted samples were analyzed via ICP-OES to determine the final concentration of Th^4+^_(*aq*)_ ([Th]*_f_*).

**Separation of thorium from actinium-225 via selective precipitation (large scale ~*10 g of dissolved thorium metal*).** To mimic the scale of current ^225^Ac production in the United States, a solution containing Th^4+^_(*aq*)_, ^225^Ac^3+^_(*aq*),_ and ^139^Ce*^n^*^+^_(*aq*)_ was prepared by dissolving Th metal (~10 g) in HNO_3(*aq*)_ (15.4 M) and (NH_4_)_2_SiF_6(*aq*)_ (ca. 7.5 mM) following the procedure given above with ^225^Ac^3+^_(*aq*)_ (ca. 8 μCi, 296 kBq) and ^139^Ce*^n^*^+^_(*aq*)_ (ca. 1.5 μCi, 55.5 kBq). After filtration to remove undissolved thorium residues (Durapore^®^ 0.22 µm PVDF–250 mL, Stericup^®^ Quick Release), the mass of the resulting filtrate was recorded and aliquots (3 × 1.00 mL) were taken and gravimetrically diluted (5.00 mL) with HNO_3(*aq*)_ (0.1 M) for γ-spectrometry to determine the initial concentrations of ^225^Ac^3+^_(*aq*)_ and ^139^Ce*^n^*^+^_(*aq*)_ ([Ac]*_i_* and [Ce]*_i_*, respectively). Additional aliquots (3 × 50 µL) were taken and diluted with HNO_3(*aq*)_ (0.1 M) by a factor of 10,000. These diluted samples were analyzed via ICP-OES to determine the initial concentration of Th^4+^_(*aq*)_ ([Th]*_i_*).

To the remaining solution (ca. 145 mL, 42 mmol Th^4+^_(*aq*)_) either KNO_3(*s*)_ (25 g, 250 mmol) or CsNO_3(*s*)_ (25 g, 130 mmol) was added. The resulting suspensions were mixed (400–550 rpm, 1 h) and the white precipitate which formed was removed by filtration (250 mL Durapore^®^ 0.22 µm PVDF, Stericup^®^ Quick Release). Aliquots of the filtrate (3 × 1.00 mL) were taken, weighed, diluted (to 5.00 mL) with HNO_3(*aq*)_ (0.1 M), and analyzed via γ-spectrometry to determine the final concentrations of ^225^Ac^3+^_(*aq*)_ and ^139^Ce*^n^*^+^_(*aq*)_ ([Ac]*_f_* and [Ce]*_f_*, respectively). Additional aliquots (3 × 50 µL) were taken and diluted with HNO_3(*aq*)_ (0.1 M) by a factor of 100 for analysis via ICP-OES to determine the final concentration of Th^4+^_(*aq*)_ ([Th]*_f_*). Each recovered precipitate on the filter was then washed consecutively with 3 aliquots (3 × 20 mL) of either KNO_3(*aq*)_ (2 M in 15.4 M HNO_3(*aq*)_) or CsNO_3(*aq*)_ (1 M in 15.4 M HNO_3(*aq*)_) depending on the salt used in the initial precipitation (see Figure S10). Each wash was collected, and quantitative analysis was done in triplicate via γ-spectrometry and ICP-OES to determine the final concentrations of ^225^Ac^3+^_(*aq*)_ and ^139^Ce*^n^*^+^_(*aq*)_ ([Ac]*_f_* and [Ce]*_f_*) or Th^4+^_(*aq*)_ ([Th]*_f_*), respectively (see Figure S2).

Percent recovery (Equation (4)) of either ^225^Ac^3+^_(*aq*)_ or ^139^Ce*^n^*^+^_(*aq*)_ from a given precipitation was determined by the summation of the percent of total activity recovered from each process step (cf. Figure S2), $p_{step}$ (e.g., filtration, wash 1, wash 2, and wash 3):

(4) $$Percent\;Recovery=\;\sum p_{step}$$

The percent recovery of a given radionuclide following a selective precipitation was calculated by:

(5) pstep=100×∑fnAfgfn×gstep∑inAigin×gb

From an initial solution containing Th^4+^_(*aq*)_ and other radionuclides (e.g., ^225^Ac^3+^_(*aq*)_, ^139^Ce*^n^*^+^_(*aq*)_, etc.), the mass of solution (*g_i_*) and activity of a given radionuclide (*A_i_*) was determined for *n* aliquots (typically *n* = 3). The average activity of the radionuclide in the bulk initial solution was then calculated from the average activity per unit mass determined from *n* aliquots and multiplied by the mass of the bulk initial solution (*g_b_*). Following a process step (e.g., filtration or wash), the mass (*g_f_*) and activity of the radionuclide (*A_f_*) was determined for *n* aliquots of the post-process solution. The average activity of a given radionuclide of the post-process solution was then calculated from its average activity per unit mass multiplied by the mass of the total post-process solution (*g_step_*).

**Separation of thorium from actinium-225 via selective precipitation (*10 g scale* *with dissolved thorium metal and irradiated target material*).** To gain an understanding of the impact of co-produced isotopes on the Th/Ac separation at medically relevant production scales, a solution containing Th^4+^_(*aq*)_ was prepared by dissolving Th^0^_(*s*)_ (19.99 g) in HNO_3(*aq*)_ (15.4 M, 300 mL) and (NH_4_)_2_SiF_6(*aq*)_ (ca. 7.5 mM) following the procedure given above and spiked with irradiated thorium target material (<1 mL in 15.4 M HNO_3(*aq*)_) and ^225^Ac^3+^_(*aq*)_. The irradiated thorium material was added such that adequate counting statistics for dominant co-produced isotopes could be attained. Benchmarked to ^103^Ru (t_1/2_ = 39.247d, 497.085 keV, 0.910 branching ratio), this was typically ca. 0.02 μCi (0.74 kBq) ^103^Ru_(*aq*)_, depending on elapsed time from end-of-bombardment (EOB). After filtration to remove undissolved thorium, aliquots were taken for both ICP-OES and γ-spectrometric analysis as described above. The remaining dissolved material was split into two portions (123 mL and 123 mL) to be precipitated by either KNO_3(*s*)_ (22 g, 218 mmol) or CsNO_3(*s*)_ (21 g, 108 mmol), according to the procedure described above. The mass of each resulting filtrate was recorded, and aliquots (3 × 1.00 mL) were taken gravimetrically and diluted (5.00 mL) with HNO_3(*aq*)_ (0.1 M) for γ-spectrometry to qualitatively evaluate the recovery of ^225^Ac^3+^_(*aq*)_ and co-produced isotopes. Additional aliquots (3 × 50 µL) of each filtrate were taken and diluted with HNO_3(*aq*)_ (0.1 M) by a factor of 100. These diluted samples were analyzed via ICP-OES to determine the concentration of Th^4+^_(*aq*)_ ([Th]*_f_*). The filtered solid from each precipitation was washed (3 × 20 mL) with either KNO_3(*aq*)_ (2 M) or CsNO_3(*aq*)_ (1 M) in HNO_3(*aq*)_ (15.4 M), and the washes were individually collected and analyzed by ICP-OES and γ-spectrometry in the manner described above.

**Isolation of single crystal thorium nitrate salts**. Stock solutions of Th^4+^_(*aq*)_ (ca. 0.5 M) were prepared by dissolving Th(NO_3_)_4_·*x*H_2_O (6 g, 12.5 mmol) in volumetric flasks (25 mL) with HNO_3(*aq*)_ (6 M, 8 M or 15.4 M).

**(NH_4_)_2_Th(NO_3_)_6_**. NH_4_NO_3(*s*)_ (3.2 g, 40 mmol) was added to the thorium stock solution (0.5 M, 10.0 mL, 5 mmol) in HNO_3(*aq*)_ (15.4 M). The resulting solution was mixed vigorously (800 rpm, 5 min). Colorless crystals formed after 3 days in an uncapped vial at room temperature.

**K_3_[Th(NO_3_)_6_](NO_3_)(HNO_3_)_3_·3H_2_O**. An aliquot of a Th^4+^_(*aq*)_ stock solution (0.5 M, 5.0 mL, 2.5 mmol) in HNO_3(*aq*)_ (8 M) was added to a glass scintillation vial (20 mL, Wheaton) followed by KNO_3(*aq*)_ (2 M, 5.0 mL, 10 mmol) in HNO_3(*aq*)_ (8 M). Colorless crystals formed after 2–3 days in an uncapped vial at room temperature.

**Rb_2_Th(NO_3_)_6_.** An aliquot of the stock Th^4+^_(*aq*)_ solution (5.0 mL, 2.5 mmol) in HNO_3(*aq*)_ (6 M) was added to a glass scintillation vial (20 mL, Wheaton) followed by RbNO_3(*aq*)_ (1.5 M, 5.0 mL, 7.5 mmol) in HNO_3_ (6 M). Colorless crystals formed after 2–3 days in an uncapped vial at room temperature.

**Cs_2_Th(NO_3_)_6_.** An aliquot of the stock Th^4+^_(*aq*)_ solution (5.0 mL, 2.5 mmol) in HNO_3(*aq*)_ (6 M) was added to a glass scintillation vial (20 mL, Wheaton) followed by CsNO_3(*aq*)_ (1.5 M, 5.00 mL, 7.5 mmol) in HNO_3_ (6 M). Colorless crystals formed after 2–3 days in an uncapped vial at room temperature.

**Single Crystal X-ray Diffraction Measurements**. A series of X-ray diffraction measurements were conducted on single crystals of (NH_4_)_2_Th(NO_3_)_6_, Rb_2_Th(NO_3_)_6_, Cs_2_Th(NO_3_)_6_, K_3_[Th(NO_3_)_6_](NO_3_)(HNO_3_)_3_·3H_2_O. For each thorium nitrate salt, single crystals suitable for X-ray analysis were obtained by decanting the mother liquor from colorless crystals, washing (3 × 5 mL) with HNO_3(*aq*)_ (15.4 M), and air drying (24 h) prior to X-ray analysis.

All crystals were submerged in mineral oil and mounted on a MiTeGen Micromount^TM^ (Ithaca, NY, USA). Low-temperature (100 K) X-ray diffraction data were collected using a Rigaku XtaLab Mini II (The Woodlands, TX, USA) two-circle diffractometer using a Mo Kα (λ = 0.71073 Å) fine-focus sealed X-ray tube source and a graphite monochromator detector. The structures were solved using the Olex2 software package and SHELXT structure solution program by Intrinsic Phasing [80,81]. Data were then refined with the XL refinement package using Least Squares minimization [82]. The final unit cell parameters obtained after structure resolution were in excellent agreement with those reported previously (Table S1) [52,83].

**Powder X-ray Diffraction Measurements.** Powder X-ray diffraction studies were completed at room temperature on a Bruker D2 Phaser benchtop X-ray diffractometer using a Cu source (Wavelength = 1.54059 Å) and a LYNXEYE XE-T detector (both from Billerica, MA, USA). The samples were contained in Dow Corning^®^ high-vacuum grease in a Bruker silicon wafer airtight low-background specimen-sample holder. Data analysis was performed in MDI JADE^®^ XRD pattern processing software (PDF4+ 2023), and phase comparison was carried out using the PDF4+ reference database. Background subtraction and data smoothing were performed using the CrystalDiffract program within the CrystalMaker software suite (Version 10).

**K_3_[Th(NO_3_)_6_](NO_3_)(HNO_3_)_3_·3H_2_O**. Data acquisition was completed from 10 to 80° 2θ over a period of 30 min with a step size of 0.02° and a stage rotation of 15.0 rotations/min.

**Rb_2_Th(NO_3_)_6_.** Data acquisition was completed in a range of 10–80° 2θ over a period of 30 min with a step size of 0.02° and a stage rotation of 15.0 rotations/min.

**Cs_2_Th(NO_3_)_6_.** Data were collected over a range of 5–65° 2θ over a period of 6 h with a step size of 0.02° and a stage rotation of 15.0 rotations/min.

## 4. Conclusions

Selective precipitation as a separation technique was investigated for its application in medical isotope purification, specifically the precipitation of large amounts of Th^4+^ from dissolved irradiated metal relevant to production of ^225^Ac. Automation of small-scale Th^4+^ precipitation experiments allowed for rapid exploration of the impact of the cation identity, precipitation agent stoichiometry, and HNO_3(*aq*)_ concentration on the ability of four nitrate salts to precipitate Th^4+^ from HNO_3(*aq*)_ solutions. The results from the automated experiments directed the study of thorium–actinium separation conditions utilizing thorium solutions prepared in an optimized nitric acid dissolution procedure. These studies demonstrated that KNO_3(*s*)_ and CsNO_3(*s*)_ were both promising candidates for the separation of Th^4+^_(*aq*)_ from ^225^Ac^3+^_(*aq*)_ via selective precipitation. Finally, we showed that 99.9% of in-going thorium could be removed from ^225^Ac^3+^_(*aq*)_ on the scale of tens of grams of dissolved thorium metal in the presence of co-produced isotopes using KNO_3(*s*)_ as a thorium precipitation agent. We anticipate that the use of KNO_3(*s*)_ has the potential to increase the access to ^225^Ac from irradiated thorium targets by speeding up chemical separations, increasing the robustness of the thorium removal process, and decreasing the amount of radioactive liquid waste produced compared to conventional methods—an advantage that compounds with scale. This procedure is additionally attractive because precipitations are common and represent chemical manipulations that are routinely conducted during radionuclide processing campaigns. They do not require special training or rigorous control of the chemical conditions and are compatible with remote handling techniques used in hot cells. Moreover, the entire thorium removal process can be completed in two hours—substantially faster than the chromatographic separation methods often used today. Investigations on the impact of Th^4+^ removal via selective precipitation with KNO_3(*s*)_ on downstream processing steps that further purify ^225^Ac^3+^ are ongoing. We also hope that the fast and easy method of selective precipitation for separations inspires others to utilize this often-overlooked method, particularly in terms of considering how countercations can play an important role in actinide speciation via selective precipitation.

## 5. Patents

Portions of this work are included in the United States Patent Application No. 63/854,065, filed on 30 July 2025.

## Figures and Tables

**Table 1:** Precipitation yield (%) of Th^4+^_(*aq*)_, ^225^Ac^3+^_(*aq*)_, and ^139^Ce*^n^*^+^_(*aq*)_ following ^nat^Th^0^_(*s*)_ (ca. 10 g) dissolution, precipitation, filtration and precipitate washing. Quantities for each element are summed across the filtrate and three wash fractions.

Precipitation Agent	Total Precipitation Yield (%)		
	Th^4+^_(*s*)_	^225^Ac^3+^_(*aq*)_	^139^Ce*^n^*^+^_(*aq*)_
KNO_3(*s*)_	95.3 ± 0.2	2.6 ± 2.0	21.1 ± 2.7
CsNO_3(*s*)_	98.39 ± 0.02	22.4 ± 0.3	52.8 ± 1.6

## Data Availability

The original contributions presented in this study are included in the article and Supplementary Information.

## Supplementary Materials

The following supporting information can be downloaded at: https://www.mdpi.com/article/10.3390/molecules31122144/s1, Supporting figures for separations chemistry Figure S1: Qualitative comparison of the gamma spectra of (*green*) dissolved thorium solution spiked with ^225^Ac^3+^_(*aq*)_ and ^139^Ce^n+^_(*aq*)_ in HNO_3(*aq*)_ (ca. 15.4 M) and (*black*) filter (Durapore^®^ 0.22 µm PVDF, 250 mL Stericup^®^ Quick Release) used to capture fines leftover from the dissolution of thorium metal spiked with ^225^Ac^3+^_(*aq*)_ and ^139^Ce^n+^_(*aq*)_. Figure S2: The precipitation yields of Th^4+^_(*aq*)_, ^225^Ac^3+^_(*aq*)_, and ^139^Ce^n+^_(*aq*)_ from solutions with Th^4+^_(*aq*)_ freshly dissolved (ca. 10 g Th^0^_(*s*)_) in HNO_3(*aq*)_ (15.4 M) following addition of either KNO_3(*s*)_ (6 molar equivalents with respect to Th^4+^_(*aq*)_) or CsNO_3(*s*)_ (3 molar equivalents with respect to Th^4+^_(*aq*)_) as a function of process step. Figure S3: Gamma spectra of (*black*) dissolved thorium metal spiked with irradiated target material, ^225^Ac_(*aq*)_, ^139^Ce_(*aq*)_ in HNO_3(*aq*)_ (15.4 M) and (*magenta*) the resulting filtrate post-precipitation with CsNO_3(*s*)_. Figure S4: The precipitation yields of Th^4+^ and ^225^Ac^3+^ from aqueous nitric acid (15.4 M) solutions of freshly dissolved Th^4+^_(*aq*)_ (ca. 1 g Th^0^_(*s*)_) spiked with ^225^Ac^3+^_(*aq*)_ and irradiated thorium target material (radiotracer concentration) as a function of precipitating agent identity and initial Th^4+^_(*aq*)_ concentration. KNO_3(*s*)_ and CsNO_3(*s*)_ were used at molar ratios of 6 and 3 with respect to Th^4+^_(*aq*)_. Figure S5: Qualitative comparison of the gamma spectra of aliquots of (*black traces*) thorium metal dissolved in HNO_3(*aq*)_ (15.4 M) spiked with irradiated target material and (*magenta traces*) the resulting filtrate following selective precipitation of Th^4+^ with KNO_3(*s*)_ (6 molar equivalents with respect to Th^4+^_(*aq*)_). Table S1: The space group and unit cell parameters determined from single crystals of (NH_4_)_2_Th(NO_3_)_6_, Rb_2_Th(NO_3_)_6_, Cs_2_Th(NO_3_)_6_, K_3_[Th(NO_3_)_6_](NO_3_)(HNO_3_)_3_ · 3H_2_O. Figure S6: Experimental powder X-ray diffraction pattern of the thorium precipitate obtained following addition of KNO_3_ compared to the predicted patterns of two possible products from Soderholm and co-workers and Sigmon and Burns. Figure S7: Experimental powder X-ray diffraction pattern of the thorium precipitate obtained following addition of RbNO_3_ compared to the predicted pattern of Rb_2_Th(NO_3_)_6_ from Soderholm and co-workers. Figure S8: Experimental powder X-ray diffraction pattern of the thorium precipitate obtained following addition of CsNO_3_ compared to the predicted pattern of Cs_2_Th(NO_3_)_6_ from Soderholm and co-workers. Figure S9: Photo of Los Alamos National Laboratory Super Separator. Figure S10: Block diagram of separation of thorium from actinium-225 via selective precipitation with KNO_3_ precipitating agent at large scale with timeline.

## Abbreviations

The following abbreviations are used in this manuscript:

LANL	Los Alamos National Laboratory
TAT	Targeted alpha therapy
LOD	Limit of Detection
LOQ	Limit of Quantification
ICP-OES	Inductively coupled plasma optical emission spectroscopy
