# Supplementary material for: Purification of Actinium-225 from Thorium via Selective Precipitation
Source: Molecules. 2026 Jun 18;31(12):2144. doi: 10.3390/molecules31122144 (PMC13305970; doi:10.3390/molecules31122144)
Supplement: Supplementary file 1 [file molecules-31-02144-s001.zip › molecules-4296274-supplementary.pdf]

Supporting Information

Purification of Actinium-225 from Thorium via Selective Precipitation

Steven J. Schultz [1-3], Sara L. Adelman [1,*], Guy H. Dutech [1], Michael E. Fassbender [1], Christopher D. Henning [1], Brian N. Long [1], Kristen A. Pace [1], Stosh A. Kozimor [1], Veronika Mocko [1,*], Thomas E. Shaw. [1]

Los Alamos National Laboratory (LANL), P.O. Box 1663, Los Alamos, New Mexico, 87545, USA

[2] Cyclotron Institute, Texas A&M University, 120 Spence Street, College Station, Texas, 77840, USA

[3] Department of Chemistry, Texas A&M University, 120 Spence Street, College Station, Texas, 77840, USA

* Correspondence: vmocko@lanl.gov, sadelman@lanl.gov

Contents

1. **Supporting Figures for Separations Chemistry**
2. **Crystallography Figures and Results**
3. **Los Alamos National Laboratory Super Separator**
4. **Separation of Thorium from Actinium-225 via selective precipitation at large scale**
5. **References**

1. Supporting Figures for Separations Chemistry

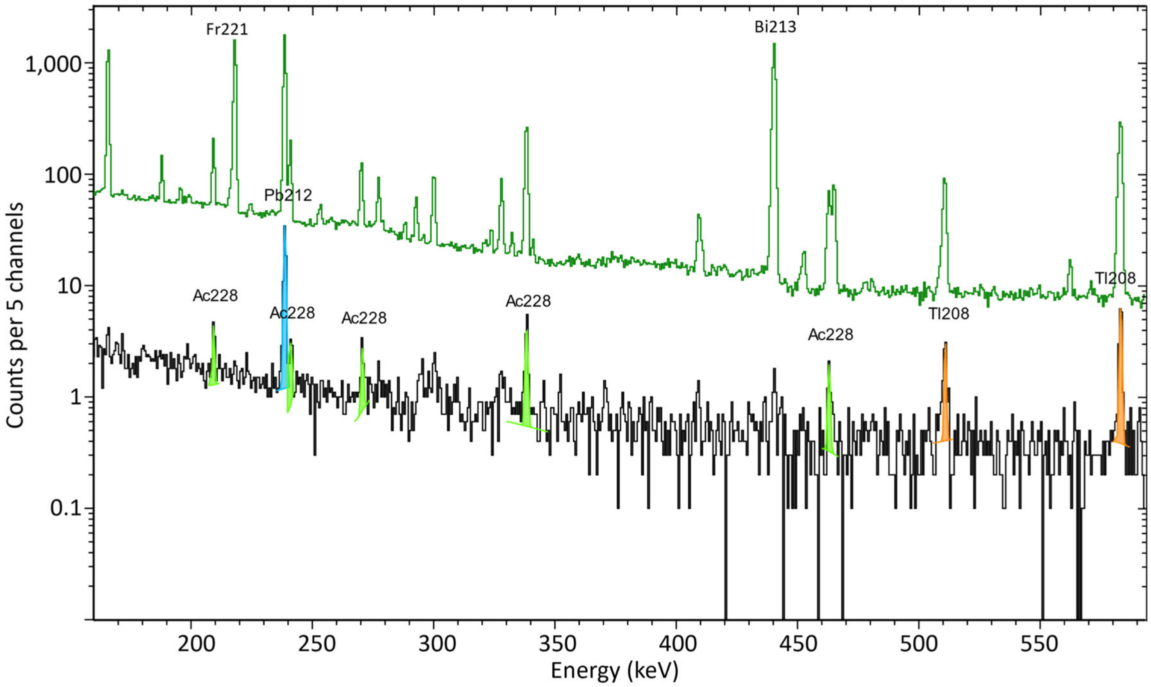

Figure S1. Qualitative comparison of the gamma spectra of (*green*) dissolved thorium solution spiked with $^{225}Ac^{3+}{}_{(aq)}$ and $^{139}Ce^{n+}{}_{(aq)}$ in $HNO_{3(aq)}$ (ca. 15.4 M) and (*black*) filter (Durapore® 0.22 µm PVDF, 250 mL Stericup® Quick Release) used to capture fines leftover from the dissolution of thorium metal spiked with $^{225}Ac^{3+}{}_{(aq)}$ and $^{139}Ce^{n+}{}_{(aq)}$.

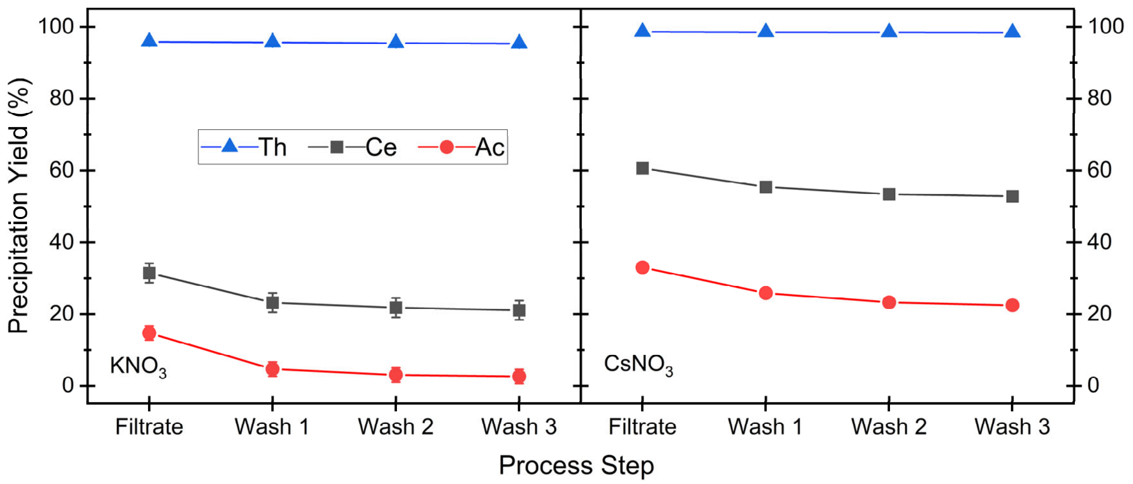

Figure S2. The precipitation yields of $Th^{4+}_{(aq)}$, $^{225}Ac^{3+}_{(aq)}$, and $^{139}Ce^{n+}_{(aq)}$ from solutions with $Th^{4+}_{(aq)}$ freshly dissolved (ca. 10 g $Th^{0}_{(s)}$) in $HNO_{3(aq)}$ (15.4 M) following addition of either $KNO_{3(s)}$ (6 molar equivalents with respect to $Th^{4+}_{(aq)}$) or $CsNO_{3(s)}$ (3 molar equivalents with respect to $Th^{4+}_{(aq)}$) as a function of process step. Wash solutions consisted of either $CsNO_{3(aq)}$ (1 M) or $KNO_{3(aq)}$ (2 M) in $HNO_{3(aq)}$ (15.4 M), according to the precipitation agent used. Lines added to guide the eye.

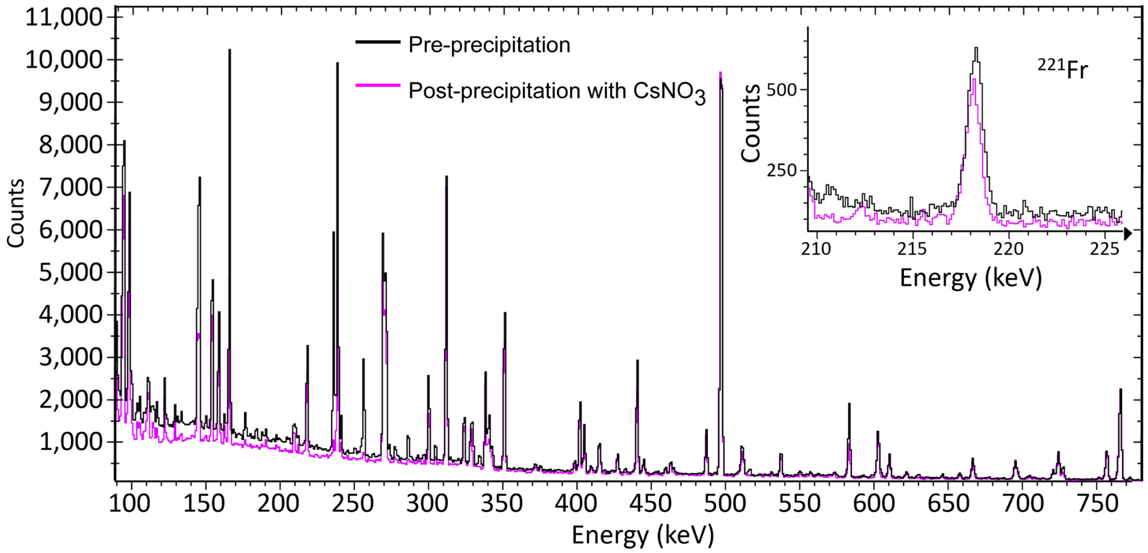

Figure S3. Gamma spectra of (*black*) dissolved thorium metal spiked with irradiated target material, $^{225}Ac_{(aq)}$, $^{139}Ce_{(aq)}$ in $HNO_{3(aq)}$ (15.4 M) and (*magenta*) the resulting filtrate post-precipitation with $CsNO_{3(s)}$.

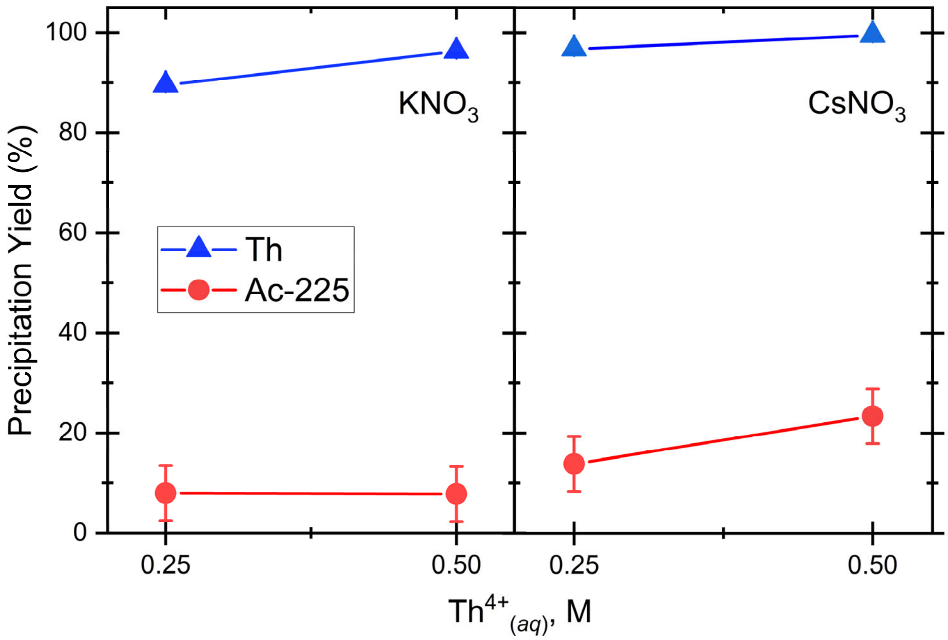

Figure S4. The precipitation yields of Th^{4+} and $^{225}Ac^{3+}$ from aqueous nitric acid (15.4 M) solutions of freshly dissolved $Th^{4+}_{(aq)}$ (ca. 1 g $Th^{0}_{(s)}$) spiked with $^{225}Ac^{3+}_{(aq)}$ and irradiated thorium target material (radiotracer concentration) as a function of precipitating agent identity and initial $Th^{4+}_{(aq)}$ concentration. $KNO_{3(s)}$ and $CsNO_{3(s)}$ were used at molar ratios of 6 and 3 with respect to $Th^{4+}_{(aq)}$. Lines between data points are to guide the eye and are not a representative fit of the data. Each data point was collected in duplicate, and the error bars represent ± 1s.

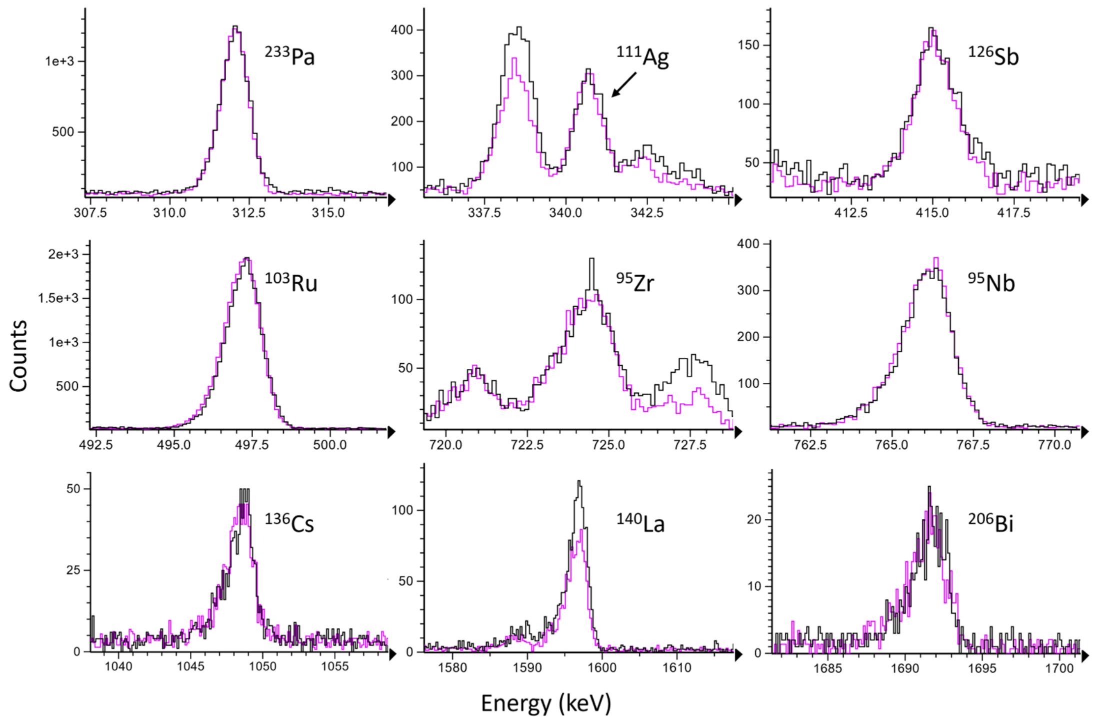

Figure S5. Qualitative comparison of the gamma spectra of aliquots of (*black traces*) thorium metal dissolved in $HNO_{3(aq)}$ (15.4 M) spiked with irradiated target material and (*magenta traces*) the resulting filtrate following selective precipitation of Th^{4+} with $KNO_{3(s)}$ (6 molar equivalents with respect to $Th^{4+}_{(aq)}$). Labelled peaks indicate isotopes that followed $^{225}Ac^{3+}_{(aq)}$ and stayed in the filtrate during the selective precipitation of thorium.

2. Crystallography Figures and Results

Table S1. The space group and unit cell parameters determined from single crystals of $(NH_4)_2Th(NO_3)_6$, $Rb_2Th(NO_3)_6$, $Cs_2Th(NO_3)_6$, $K_3[Th(NO_3)_6](NO_3)(HNO_3)_3 \bullet 3H_2O$. Data collected herein is compared against that reported previously [1]

	Space Group (this work)	Space Group (reported) [1]	a, b, c (Å) (this work)	a, b, c (Å) (reported) [1]	α, β, γ (°) (this work)	α, β, γ (°) (reported) [1]
$(NH_4)_2Th(NO_3)_6$	*P2₁/n*	*P2₁/n*	8.2420(1)	8.2653(5)	90	90
			6.8345(1)	6.8573(4)	91.384(1)	91.3982(8)
			12.8998(2)	12.9515(8)	90	90
$Rb_2Th(NO_3)_6$	*P2₁/n*	*P2₁/n*	8.2944(6)	8.3236(4)	90	90
			6.8575(6)	6.8887(4)	92.209(6)	92.2130(6)
			12.8165(8)	12.8538(7	90	90
$Cs_2Th(NO_3)_6$	*P2₁/n*	*P2₁/n*	8.0957(2)	8.131(2)	90	90
			7.1618(2)	7.197(1)	90.760(2)	90.80(3)
			13.3410(3)	13.396(3)	90	90
$K_3[Th(NO_3)_6](NO_3)(HNO_3)_3 \bullet 3H_2O$	*R3c*	*R3c*	15.6589(5)	15.6745(4	90	90
			15.6589(5)	15.6745(4	90	90
			19.1622(6)	19.1893(4	120	120

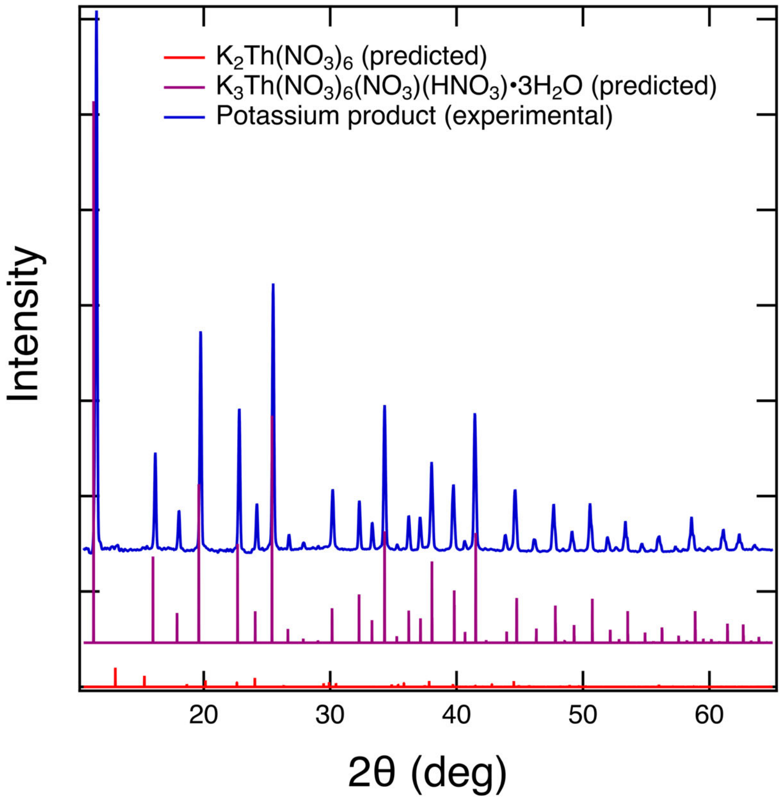

Figure S6. Experimental powder X-ray diffraction pattern of the thorium precipitate obtained following addition of KNO_3 compared to the predicted patterns of two possible products from Soderholm and co-workers and Sigmon and Burns [1,2].

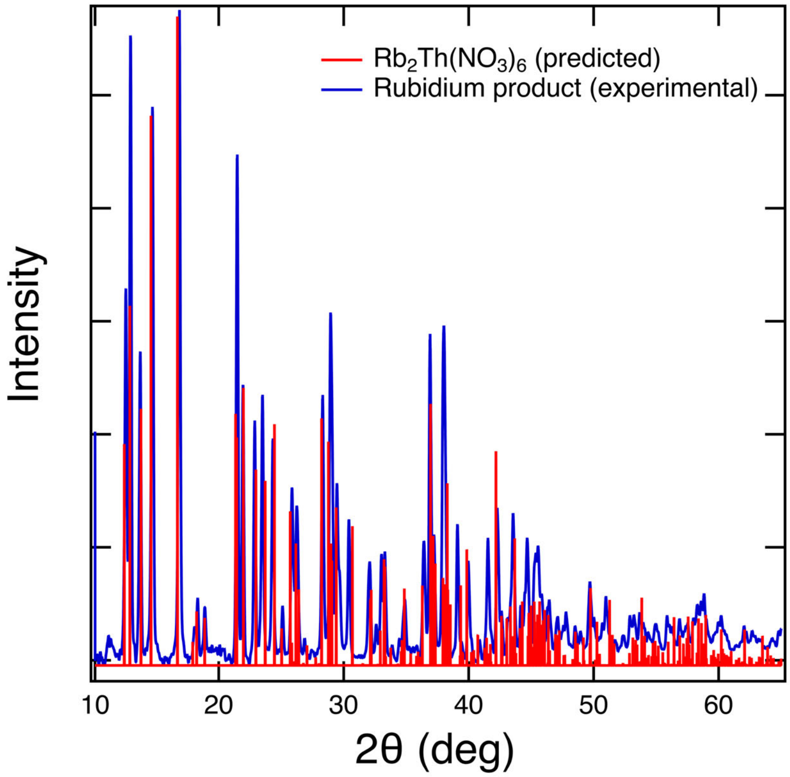

Figure S7. Experimental powder X-ray diffraction pattern of the thorium precipitate obtained following addition of $RbNO_3$ compared to the predicted pattern of $Rb_2Th(NO_3)_6$ from Soderholm and co-workers [1].

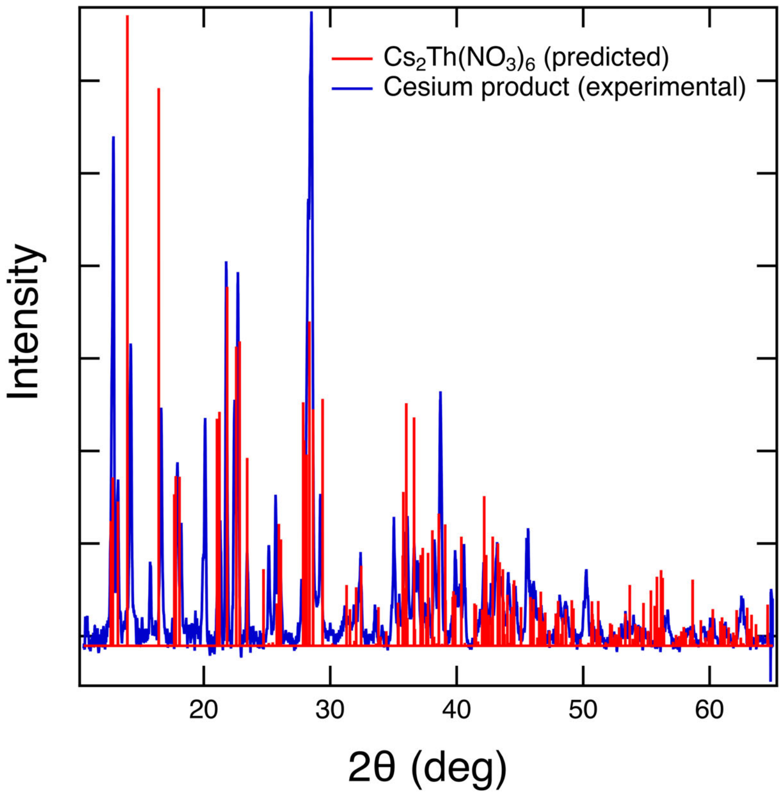

Figure S8. Experimental powder X-ray diffraction pattern of the thorium precipitate obtained following addition of $CsNO_3$ compared to the predicted pattern of $Cs_2Th(NO_3)_6$ from Soderholm and co-workers [1].

3. Los Alamos Super Separator

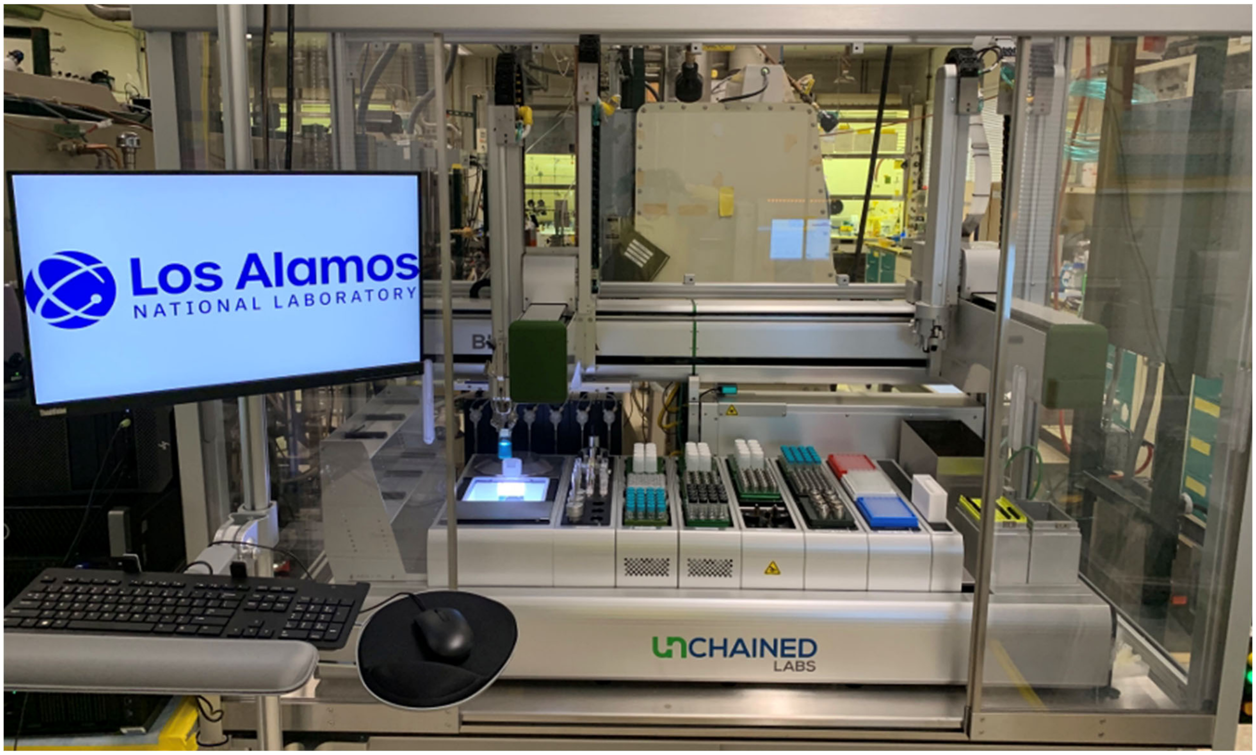

Figure S9. Photo of Los Alamos National Laboratory Super Separator.

4. Separation of thorium from actinium-225 via selective precipitation at large scale

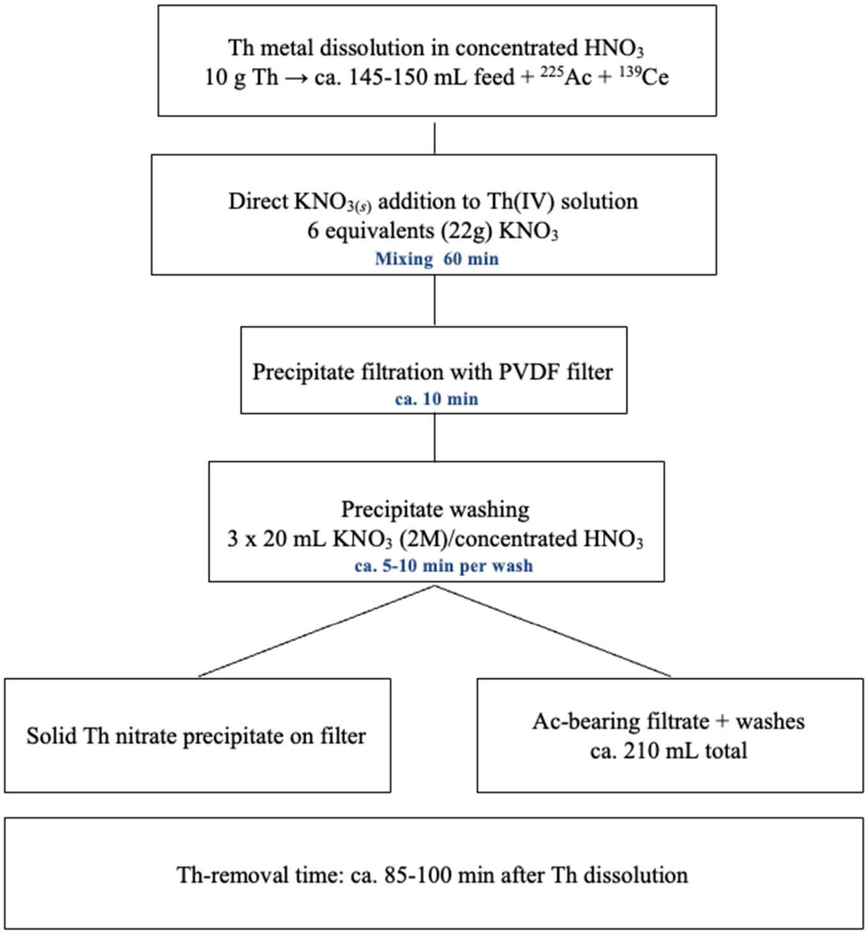

Figure S10. Block diagram of separation of thorium from actinium-225 via selective precipitation with KNO_3 precipitating agent at large scale with timeline.

5. References

1. Jin, G. B.; Lin, J.; Estes, S. L.; Skanthakumar, S.; Soderholm, L. Influence of Countercation Hydration Enthalpies on the Formation of Molecular Complexes: A Thorium–Nitrate Example. *Journal of the American Chemical Society* **2017**, *139* (49), 18003-18008. DOI: 10.1021/jacs.7b09363.
2. Sigmon, G. E.; Burns, P. C. Crystal chemistry of thorium nitrates and chromates. *Journal of solid-state chemistry* **2010**, *183* (7), 1604-1608. DOI: 10.1016/j.jssc.2010.04.042.
